# Detection and enumeration of Lak megaphages in microbiome samples by endpoint and quantitative PCR

**DOI:** 10.1016/j.xpro.2021.101029

**Published:** 2022-01-07

**Authors:** Marco A. Crisci, Paula M. Corsini, Nicola Bordin, Lin-Xing Chen, Jillain F. Banfield, Joanne M. Santini

**Affiliations:** 1 Department of Structural and Molecular Biology, Division of Biosciences, University College London, London, UK; 2 Quadram Institute Bioscience, Norwich Research Park, Norwich, UK; 3 Department of Earth and Planetary Science, University of California Berkeley, Berkeley, CA, USA; 4 Innovative Genomics Institute, University of California Berkeley, Berkeley, CA, USA; 5 The University of Melbourne, Melbourne, VIC, Australia

**Keywords:** Health Sciences, Immunology, Microbiology, Molecular Biology

## Abstract

Lak megaphages are prevalent across diverse gut microbiomes and may potentially impact animal and human health through lysis of *Prevotella*. Given their large genome size (up to 660 kbp), Lak megaphages are difficult to culture, and their identification relies on molecular techniques. Here, we present optimized protocols for identifying Lak phages in various microbiome samples, including procedures for DNA extraction, followed by detection and quantification of genes encoding Lak structural proteins using diagnostic endpoint and SYBR green-based quantitative PCR, respectively.

For complete details on the use and execution of this protocol, please refer to Crisci et al., (2021).

## Highlights

• Optimized DNA extraction procedure for use with various microbiome samples
• Amplification of Lak phage structural protein genes for detection and sequencing
• Quantification of major capsid protein genes to estimate Lak phage abundance

## Graphical abstract

**Figure:**
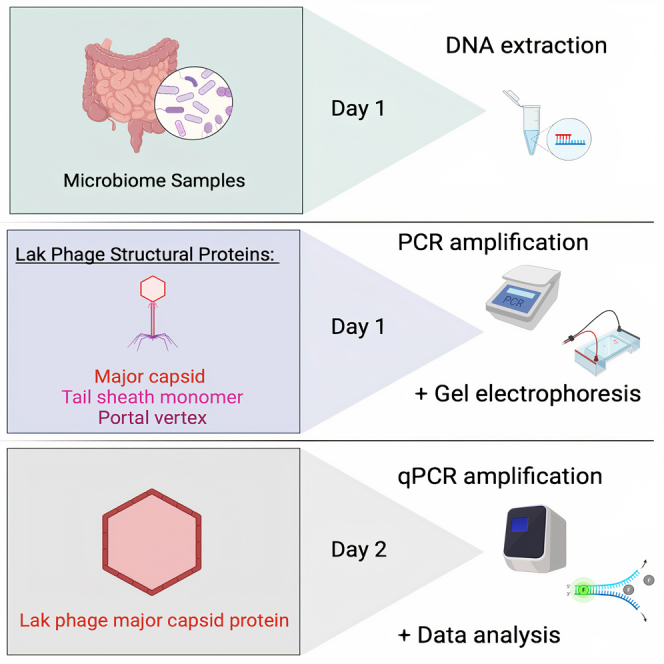

## Before you begin

The protocol below describes specific steps for processing and screening fecal, digesta and mucosal samples for Lak phages, but can be adapted for different sample types. We have also implemented the protocol for use with liquid samples (e.g., jejunal and rumen fluid) and mucosal brushes suspended in sodium-magnesium (SM) buffer.

### Sample collection and storage

**Timing: 30 min–2 h**

1. Avoid cross-contamination between samples and environmental contaminants.
  a. Change gloves between samples.
  b. Use clean, sterile collection equipment.
    i. For mucosal samples, dissection utensils can be sterilized in 100% ethanol.
    ii. For fecal/digesta samples, use sterile, disposable equipment.
  c. Collect fecal samples from the centre of the stool where possible.

**CRITICAL:** Use appropriate personal protective equipment and take care when collecting biological samples which may contain pathogens.

2. Transport and storage.
  a. Samples should be immediately flash-frozen on dry ice or liquid nitrogen on collection.
    i. If flash-freezing is not possible, transfer samples at 4°C or at room temperature (20°C–22°C)
  b. Samples should be stored at −80°C until analysis, or analyzed immediately.
    i. Thaw samples to room temperature (20°C–22°C) before DNA extraction.

### Prepare stocks of oligonucleotide primers

**Timing: 30 min**

3. Order or synthesize primers detailed in Table 1.

**Table 1:** Lak phage PCR and qPCR primer pairs

Use	Application	Primer pair	Sequence	Target	Product size (bp)
PCR	General Lak phage diagnostics/ amplicon sequencing	LakMC581-F	5′-GGAGTCATACGAACACCAGAAGT-3′	Major capsid protein	473
		LakMC1053-R	5′-GTAGTTCTTACACTTCACGCTCCTC-3′		
PCR	General Lak phage diagnostics/amplicon sequencing	LakPV767-F	5′-CATGGTCAACAGGTATGTATGG-3′	Portal vertex protein	495
		LakPV1261-R	5′-CCTCTCGTGTTATACTTGCATCA-3′		
PCR	General Lak phage diagnostics/amplicon sequencing	LakTS3039-F	5′-CTTCCATCTAAGAGACAGTTTGA-3′	Tail sheath monomer	689
		LakTS3781-R	5′-CTTCCATCTAAGAGACAGTTTGA-3′		
PCR	660 kbp Lak phage diagnostics/amplicon sequencing	LakHMC185-F	5′-CTCTTAACGAGGATGCACAGT-3′	Major capsid protein	799
		LakHMC984-R	5′-ACCTGCACCGGTATAACCAA-3′		
qPCR	General Lak phage quantification	LakMCP683-F	5′-CAACCAAGAGCGAACAAACGAG-3′	Major capsid protein	121
		LakMC803-R	5′-TAACAGACCTTCAGAAACAGTGGG-3′		
qPCR	660 kbp Lak phage quantification	LakHMC224-F	5'-GGTGGCGTTTACAGCCCATA-3′	Major capsid protein	197
		LakHMC421-R	5'-CAACGGTCTTAGCAGCAACCT-3′		

Primers detailed in this table were designed by Crisci et al. (2021).
4. Prepare stocks. For lyophilized primers:
  a. Centrifuge for 30 s at 13,000 × *g*.
  b. Resuspend in sterile Tris-EDTA (TE) buffer to stock concentration (e.g., 100 μM).
  c. Vortex for 10 min at maximum speed.
  d. Centrifuge briefly.
5. Dilute primers to working concentration of 7 μM with nuclease-free water.
  a. Centrifuge briefly.
  b. Store both stock and working solutions at −20°C.

## Key resources table

**Table:** 

REAGENT or RESOURCE	SOURCE	IDENTIFIER
**Biological samples**		

Microbiome samples (e.g., feces)	Acquired by protocol users	N/A

**Chemicals, peptides, and recombinant proteins**		

Trizma® Base	Sigma-Aldrich	Cat. No. T6066
Hydrochloric Acid (HCl; 37%)	VWR	Cat. No. 20252.290
Ethylenediaminetetraacetic acid disodium salt dihydrate (EDTA-Na_2_; 372.24 g/mol)	Sigma-Aldrich	Cat. No. E5134
Sodium hydroxide pellets (NaOH; 98.5%–100.5%)	VWR	Cat. No. 28244.262
Deoxynucleotide set (including 100 mM each: TTP in H_2_O, dCTP (pH 7), dGTP (pH 7) dATP (pH 7)).	Sigma-Aldrich	Cat. No. DNTP100A

**Critical commercial assays**		

QIAquick PCR purification kit	Qiagen	Cat. No. 28106
QIAquick gel extraction kit	Qiagen	Cat. No. 28706
QIAamp PowerFecal Pro DNA kit	Qiagen	Cat. No. 51804
QuantiNova SYBR green PCR kit (including master mix, nuclease-free water, ROX passive reference dye and template dilution buffer)	Qiagen	Cat. No. 208054
BIOTAQ^TM^ DNA polymerase kit (including polymerase, NH_4_ reaction buffer, and MgCl_2_)	Bioline	Cat. No. BIO-21060

**Deposited data**		

Results using this method	Crisci et al. (2021)	https://www.sciencedirect.com/science/article/pii/S2589004221008439

**Oligonucleotides**		

See Table 1 for Lak qPCR and PCR primers	Crisci et al. (2021)	https://www.sciencedirect.com/science/article/pii/S2589004221008439

**Software and algorithms**		

JMP® Pro 14.1	SAS Institute Inc., NC, USA, 2019	https://www.jmp.com/en_gb/home.html

**Other**		

OrionTM 3-Star benchtop pH meter	Thermo Scientific^TM^	Cat. No. 13-644-928
NanoDrop^TM^ 2000 spectrophotometer	Thermo Scientific^TM^	Cat. No. ND2000LAPTOP
Piko PCR plate, 96-well	Thermo Scientific^TM^	Cat. No. SPL0961
Mastercylcler® nexus gradient PCR thermal cycler	Eppendorf	Cat No. 6331000041

## Materials and equipment

### Tris-EDTA (TE) buffer

Preparation of TE buffer is recommended for storage of oligonucleotide primers. This can be prepared as follows:

Prepare Tris-HCl (1 M, pH 8):
  Dissolve 121.14 g Trizma® base (121.14 g/mol) in 800 mL deionized water.
  Using a pH meter, adjust to pH 8 by adding HCl (37%) as required.
  Adjust volume to 1000 mL with deionized water.
Prepare EDTA (0.5 M, pH 8)
  Dissolve 186.12 g EDTA-Na_2_ (372.24 g/mol) in 800 mL deionized water.
  Using a pH meter, adjust to pH 8 by adding NaOH pellets (**≥** 98.5%) as required.
  Adjust volume to 1000 mL with deionized water.
Then, prepare the TE buffer (pH 8):

**Table:** 

Reagent	Final concentration	Volume to add for 100 mL
Deionized water	n/a	98.8 mL
Tris-HCl (1 M, pH 8)	10 mM	1 mL
EDTA (0.5 M, pH 8)	1 mM	0.2 mL

Sterilize by autoclaving (110°C for 15 min), or filtration (0.22 μm membrane filter), and store at room temperature (20°C–22°C).

***Alternatives:*** Purchase pre-prepared TE buffer (e.g., Invitrogen^TM^, Cat. no. 12090015)

### Deoxynucleotide triphosphates (dNTPs)

Preparation of dNTPs is essential for the proposed PCR assay. Prepare solution as follows:

**Table:** 

Reagent	Final concentration	Volume to add for 100 μL
Nuclease-free water	n/a	60 μL
dATP (100 mM, pH 7)	10 mM	10 μL
TTP (100 mM, in H_2_O)	10 mM	10 μL
dCTP-Na_2_ (100 mM, pH 7)	10 mM	10 μL
dGTP-Na_3_ (100 mM, pH 7)	10 mM	10 μL

Store at −20°C.

***Alternatives:*** Purchase pre-prepared dNTPs (e.g., Thermo Scientific^TM^, Cat. no. R0181)

## Step-by-step method details

### DNA extraction from microbiome samples

**Timing: 1 h**

Validated procedure for DNA extraction using PowerFecal Pro DNA kit (Qiagen, Hilden, Germany) on a range of microbiome samples, including feces/solid digesta, mucosal tissues and fluid digesta. Follow manufacturer instructions with adaptations as below:

1. Lysis.
  a. Mix solid samples in collection tubes with a sterile spatula.
  b. Place approximately 0.25 g of solid sample in a bead tube with 800 μL lysis buffer.
    i. For liquid samples, use 200 μL of sample with 600 μL lysis buffer.
  c. Heat tubes at 65°C for 10 min, using a water bath or heat block.
    ***Note:*** Heating will aid disassembly of the phage capsid and the release of genetic material.
  d. Using a vortex adapter, vortex tubes for 10 min at maximum speed.

2. Inhibitor removal and washing (as specified in manufacturer instructions)

3. Elution.
  a. Use 60 μL of elution buffer and incubate spin column at room temperature (20°C–22°C) for 10 min.
    i . Centrifuge at 13,000 ×*g* for 1 min.
    ii . Once eluted, pass the same 60 μL eluate back through the same spin column (without incubation) and repeat centrifugation.

4. Measure DNA concentration by UV spectrophotometry.
5. Store DNA at −20°C until analysis.

**RECOMMENDED:** Use of filter tips when processing microbiome samples.

### Amplification of Lak phage structural protein-coding genes by PCR

**Timing: 1 h 30 min**

Amplification of Lak phage major capsid protein (MCP), portal vertex protein (PVP) and tail sheath monomer (TSM) gene fragments can be used for detection of Lak phages in biological samples. These signature genes were selected from metagenomic datasets, due to their specificity and conservation across Lak phage genomes. The general screening primers are specific to Lak phages found in humans, primates, pigs and most other animals. An additional set was designed to detect the divergent ∼660 kbp variant discovered in a racehorse microbiome (Crisci et al., 2021). Primer sequences are reported in Table 1.

6. On ice, prepare master mixes for each assay containing the following:

**Table:** 

Reagent	Final concentration per 25 μL reaction	Volume to add per 25 μL reaction
dNTPs	20 μM (of each dNTP)	0.5 μL
Forward primer (variable)	0.14 μM	0.5 μL (of 7 μM primers)
Reverse primer (variable)	0.14 μM	0.5 μL (of 7 μM primers)
MgCl_2_	3 mM	1.5 μL
NH_4_ reaction buffer	1×	2.5 μL

7. Calculate required volume of DNA to achieve 150 ng/μL in 25 μL.
8. Calculate required volume of nuclease-free water (e.g., 19.25 μL – DNA volume).
9. On ice, prepare 25 μL PCR reaction:
  a. Add water and DNA.
  b. Add 5.5 μL pre-prepared master mix.
  c. Add 0.25 μL Taq DNA Polymerase (1.25 U; BIOTAQ^TM^ DNA Polymerase, Bioline, London, UK).

***Note:*** Although this protocol has been validated with BIOTAQ^TM^ DNA Polymerase, users may adapt for other DNA polymerases or master mixes.

10. Centrifuge briefly, and place in a thermocycler programmed under the following conditions:

**Table:** 

PCR cycling conditions				
Steps	Target	Temperature	Time	Cycles
Initial Denaturation	MCP, PVP, TSM and MCP_660	96°C	30 s	1
Denaturation	MCP, PVP, TSM and MCP_660	96°C	10 s	40 cycles
Annealing	MCP	61°C	30 s	
	PVP	58°C	30 s	
	TSM	57°C	30 s	
	MCP_660	57°C	30 s	
Extension	MCP and PVP	72°C	15 s	
	TSM	72°C	20 s	
	MCP_660	72°C	25 s	
Final extension	MCP, PVP, TSM and MCP_660	72°C	10 min	1
Hold	MCP, PVP, TSM and MCP_660	4°C	thereafter	

### Endpoint analysis of PCR products

**Timing: 2 h**

11. Presence of Lak phage in microbiome DNA can be visualized using agarose gel electrophoresis.
12. Purified PCR products can be sequenced for confirmation and comparison. We recommend using either QIAquick PCR purification or gel extraction kits (Qiagen, Hilden, Germany) prior to sequencing. To maximise yield, follow manufacturer instructions with adaptations as below:
  a. Use 30 μL of elution buffer and incubate spin column at room temperature for 10 min.
  b. Centrifuge at 13,000 ×*g* for 1 min.
  c. Once eluted, pass the same 30 μL eluate back through the same spin column (without incubation) and repeat centrifugation.
13. Measure DNA concentration by UV spectrophotometry.

**CRITICAL:** PCR purification of Lak MCP gene amplicons is a prerequisite in generating standards for the following qPCR assay.

### qPCR quantification of Lak phage major capsid protein-coding gene

This protocol has been validated for use with the QuantiNova SYBR Green PCR kit (Qiagen, Hilden, Germany), and PikoReal^TM^ real-time PCR system (Thermo Fisher Scientific, MA, USA) (Crisci et al., 2021). However, the following procedure can be adapted for any SYBR green-based qPCR kit and compatible instrument. As Lak phage genomes are generally AT-rich, the MCP gene was selected due to its conservation and higher GC content (∼41%) than other genes, which provides better thermal stability in qPCR reactions.

**CRITICAL:** The use of PCR product as qPCR standard is expected to yield an efficiency over 100% (Crisci et al., 2021). Efficiencies between 90 and 110% are considered acceptable for quantification. It is advisable to calculate amplification efficiencies using standards and primers prior to qPCR quantification.

**e.g.** (−1+10^−1/slope^) × 100

### Prepare standards for qPCR

**Timing: 30 min**

Lak phage MCP gene amplicons (MCP: LakMC581-F/LakMC1053-R and MCP_660: LakHMC185-F/LakHMC984-R) are used as qPCR standards. The qPCR target regions are within the regions amplified by the endpoint PCR primers (Table 1). Once a PCR product is obtained, prepare qPCR standards as follows.

14. Prepare 9 × 1:10 serial dilutions of PCR amplicon (starting at 5 ng).
  a. In the first tube, adjust Lak major capsid PCR product to 5 ng/μL using nuclease-free water or 1× template dilution buffer.
  b. Then, add 2 μL from the first tube to 18 μL nuclease-free water or 1× template dilution buffer, mix by pipetting up and down, and discard the tip.
    i. Repeat for remaining serial dilutions. For example:

**Table:** 

Dilution order	DNA concentration (ng/μL)	Water volume (μL)	DNA volume (μL)
1	5	Variable	Variable
2	0.5	18	2
3	0.05		
4	0.005		
5	0.0005		
6	0.00005		
7	0.000005		
8	0.0000005		
9	0.00000005		

### Prepare DNA dilutions for qPCR

**Timing: 30 min**

15. Adjust sample DNA to 10 ng/μL using nuclease-free water or 1× template dilution buffer (so that 1 μL provides 10 ng DNA per 10 μL qPCR reaction).

### qPCR amplification

**Timing: 1 h**

16. Prepare master mixes for each assay containing the following:

**Table:** 

Reagent	Final concentration per 10 μL reaction	Volume to add per 10 μL reaction
Nuclease-free water	n/a	1 μL
Forward primer (variable)	0.7 μM	1 μL (of 7 μM primers)
Reverse primer (variable)	0.7 μM	1 μL (of 7 μM primers)
ROX passive reference dye	1×	1 μL∗
QuantiNova SYBR Green master mix	1×	5 μL

***∗Optional:*** ROX reference dye is used for normalization of fluorescence signals. ROX can be replaced with the same volume of nuclease-free water if not required.

17. Load 9 μL master mix into 96-well plates.
18. Load 1 μL diluted DNA:
  a. Standards (pre-prepared serial dilutions)
  b. Sample DNA (10 ng per reaction)
19. Seal plates with optical heat seals.
20. Program thermocycler according to kit instructions for quantification with SYBR green using the standard curve method.

***Note:*** A minimum of 3 technical replicates is recommended, along with use of no template controls (NTC; water in place of DNA). Melt curve analysis can be performed to identify non-specific binding.

21. Extrapolate major capsid gene quantities (ng) from standard curves (quantification cycle (Cq) vs. Log DNA dilution).
  a. Extrapolated quantities (ng) can be analysed as is, or converted to copy numbers.

***Note:*** The MCP gene is single copy. Thus, gene copy number = genome copies.

## Expected outcomes

### Expected PCR outcomes: DNA extraction

Across 169 samples, the present method for total DNA extraction yielded an average concentration of 367.1 ng μL^−1^. For mucosal samples, yields ∼1000 ng μL^−1^ were common, which are likely attributed by host DNA. For fecal samples and fresh digesta, ∼70 ng μL^−1^ to ∼400 ng μL^−1^ DNA was obtained.

### Expected PCR outcomes: endpoint PCR results

**Figure 1:**
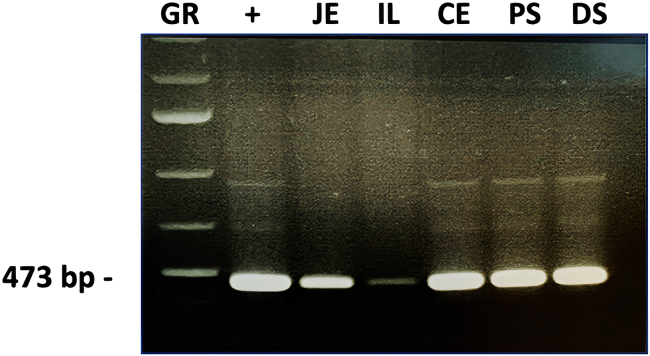
Representative Lak phage major capsid PCR products visualised by agarose gel electrophoresis **GR** = 1 kb+ GeneRuler; **+** = Swine positive control; **Lak MCP PCR amplicons from pig gastrointestinal samples = JE**=Jejunum; **IL** = Ileum; **CE** = Caecum; **PS** = Proximal spiral; **DS** = Distal spiral. See Table 1 for primer details. Data relating to this figure has been published previously by Crisci et al. (2021).

The present endpoint PCR method using phage structural proteins is effective for detection of Lak phages in a variety of microbiome samples. Figure 1 shows positive PCR results for the Lak MCP assay, derived from pig digesta samples.

### Expected qPCR outcomes

**Figure 2:**
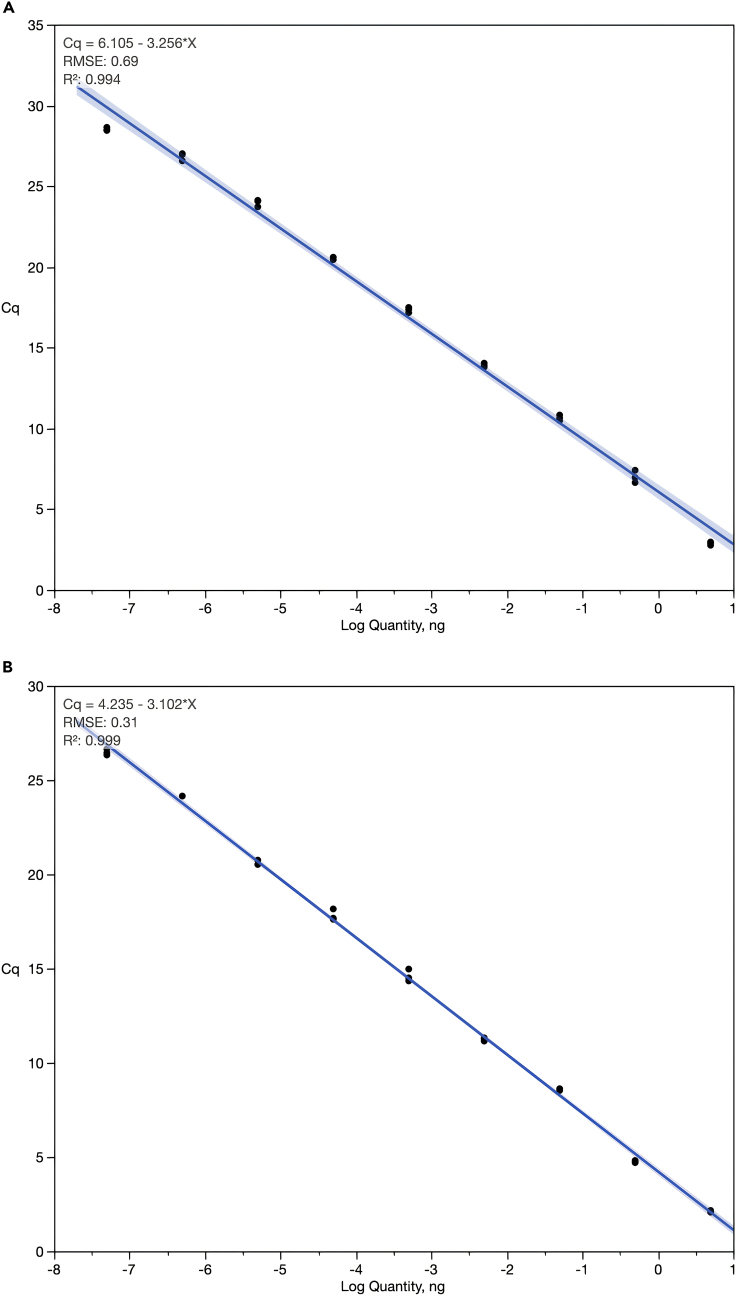
Standard curves generated using Lak phage qPCR primers Cq = Cycle of quantification; R^2^ = Coefficient of determination; RMSE = Root mean square error. Black dots represent technical replicates for each serial dilution of Lak major capsid amplicon (MCP: LakMC581-F/LakMC1053-R; MCP_660: LakHMC185-F/ LakHMC984-R): 1:10 starting at 5 ng. Primer efficiencies (E) calculated from slopes ((−1+10^−1/slope^) × 100). (A) Lak MCP amplicons used as standards with LakMC683-F/LakMC803-R primers, E = 102.8%. (B) Lak MCP_660 variant amplicons used as standards with LakHMC224-F/LakHMC421-R primers, E = 110.1%. See Table 1 for primer details. Data relating to this figure has been published previously by Crisci et al. (2021).

Figure 2 shows representative standard curves and efficiencies obtained using the proposed qPCR assays for Lak phages (based on most known genomes; Figure 2A), and for the ∼660 kbp variant (Figure 2B).

***Note:*** Acceptable qPCR primer efficiencies for reliable quantification should be between 90 and 100%. If efficiencies outside of this range are encountered, see troubleshooting.

**Figure 3:**
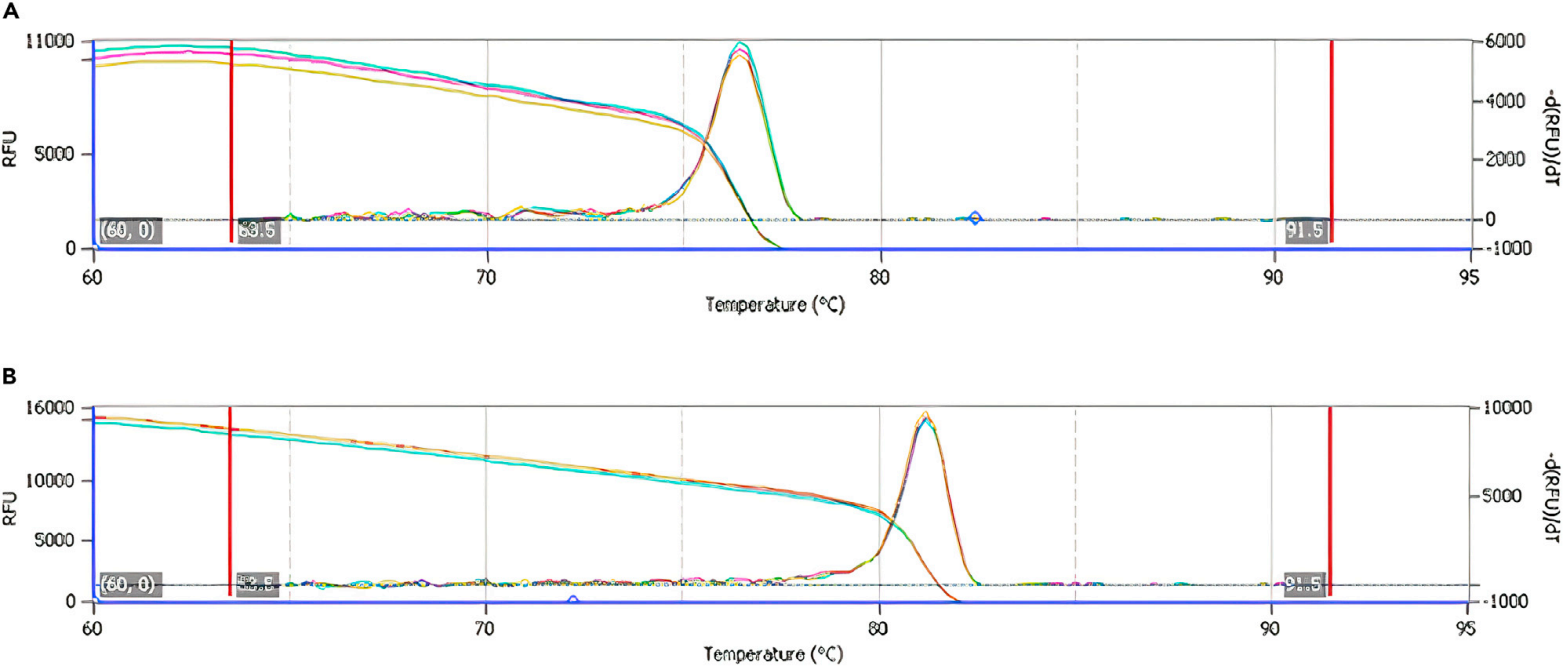
Representative melt curves for Lak qPCR primer pairs RFU = relative fluorescence units using SYBR green. Single peak generated from 3 technical replicates indicates no nonspecific binding or secondary structures. (A) Representative result shown for Lak MCP primers (LakMC683-F/LakMC803-R). (B) Representative result shown for Lak MCP_660 primers (LakHMC224-F/LakHMC421-R). See Table 1 for primer details. A version of this figure has been published previously by Crisci et al. (2021).

Figure 3 shows representative melt curves with no non-specific binding using the proposed qPCR assays for Lak phages (based on most known genomes; Figure 3A), and for the ∼660 kbp variant (Figure 3B).

Quantification results obtained using the present qPCR assays for Lak phages are published in Crisci et al. (2021). For pig gut luminal contents and mucosal tissues (using Lak_MCP) and horse faeces (using Lak MCP_660), Log copies ranged from ∼2 to ∼7.

## Quantification and statistical analysis

Depending on the users’ requirements, statistical analysis of qPCR data will vary. However, for comparison of Lak phage abundance between different treatments or sample groups, we briefly describe steps employed by Crisci et al. (2021). The proposed method for statistical analysis is compatible with JMP® Pro 14.1 (SAS Institute Inc., NC, USA, 2019), but can be adapted to virtually any statistical package.

1. Prepare data for analysis.
  a. Collate data into a table, including headings for all known variables.
  b. Calculate averages for technical replicates, and log-transform.

***Note:*** Log transformation simplifies the analysis of data that does not usually conform to normality.

2. Analyse distribution.
  a. Perform distribution analysis by group (e.g., treatment, or body site).
  b. Identify outliers and remove as required.

***Note:*** Lak phage abundance may vary beyond the normal distribution for a given population. JMP® identifies outliers as 1.5∗IQR (interquartile range).

3. Design appropriate statistical model.
  a. If feasible, use least squared mean comparisons or ANOVA.
  b. Identify covariates so that they can be accounted for in the statistical model.

***Note:*** Covariates may be factors such as sex, age, and location.

4. Means separation.
  a. When comparing >2 means, we recommend using Tukey’s HSD test. When comparing 2 means, a t test or similar can be used.

## Limitations

It should be considered that Lak phage genomes which are genetically distinct to those described by Crisci et al. (2021) and Devoto et al. (2019) may not be detected using the proposed methods. Given that qPCR assays yield efficiencies over 100% (due to use of PCR amplicons as standards), quantification results should be considered as estimations, useful for comparison purposes, rather than definitive quantities.

## Troubleshooting

### Problem 1

DNA concentration too low for PCR or sequencing (step 4).

### Potential solution

During DNA extraction, prepare multiple lysates for each sample, but pass all through the same spin column for washing and elution into one tube.

### Problem 2

False negatives (step 11).

### Potential solution 1

To confirm whether negative PCR results are true or due to the absence of Lak phages, it is recommended to use a positive control alongside unknown samples. Once Lak phages are confirmed within sample DNA, the sample should be used thereon as a positive control.

### Potential solution 2

During DNA spectrophotometry, check 260/280 and 260/230 ratios, as this can indicate contamination which may interfere with PCR. Furthermore, check that microbiome DNA is not degraded prior to use by electrophoresing 5 uL on a 1% agarose gel with 6× loading dye. If DNA is contaminated, it can be diluted or re-extracted.

### Problem 3

False positives (step 11).

Thus far, the present PCR and qPCR primers have not resulted in nonspecific binding. However, should nonspecific binding or secondary structures arise (e.g., PCR amplicon sizes not as expected), the following potential solutions can be implemented.

### Potential solution 1

It is advisable to sequence PCR products and perform a BLASTN (Altschul et al., 1990) search for confirmation. Published Lak genomes are deposited in NCBI (Crisci et al., 2021; Devoto et al., 2019).

### Potential solution 2

New primers can be designed using published Lak phage genes. Before synthesis, check primer sets for secondary structures (e.g., OligoAnalyzer; Integrated DNA Technologies Inc., Iowa, USA), and validate the assay using methods described in this protocol.

### Problem 4

Insufficient PCR product yield for sequencing (step 12).

### Potential solution

Repeat the PCR, using a larger reaction volume until a sufficient product yield is obtained.

### Problem 5

Lak phage abundance below that reliably detected using the standard curve (step 15).

### Potential solution

If Cq values for sample DNA are outside of those plotted in the standard curve (e.g., lower than highest standard dilution), the starting concentration of DNA may be increased.

### Problem 6

Inhibition of qPCR (step 15).

If Lak phages are detected in sample DNA, but are not quantifiable by qPCR, impurities in the DNA preparation may be accountable.

### Potential solution

Use further dilutions of sample DNA to reduce inhibition (e.g., reduce template DNA concentration to less than 10 ng).

### Problem 7

qPCR primer efficiencies outside of an acceptable range (step 16).

For efficiencies below 90% or above 110%, the following potential solutions can be implemented.

### Potential solution 1

Check primer concentrations are correct, prepare fresh master mix, and repeat the experiment.

### Potential solution 2

Use a larger qPCR reaction volume (e.g., greater than 10 uL), as this will decrease the propensity for pipetting errors.

## Resource availability

### Lead contact

Further information and requests for resources and reagents should be directed to and will be fulfilled by the lead contact, Joanne M. Santini (j.santini@ucl.ac.uk).

### Materials availability

This study did not generate any new unique reagents.

## Data Availability

The original/source data for figures in the paper are available (Crisci et al., 2021).
